# Quantitative Prediction of Human Immunodeficiency Virus Drug Resistance

**DOI:** 10.3390/v16071132

**Published:** 2024-07-15

**Authors:** Ekaterina A. Stolbova, Leonid A. Stolbov, Dmitry A. Filimonov, Vladimir V. Poroikov, Olga A. Tarasova

**Affiliations:** Department of Bioinformatics, Institute of Biomedical Chemistry, 10-7, Pogodinskaya Street, Moscow 119121, Russia; ekaterina.a.stolbova@yandex.ru (E.A.S.); stolbovla@gmail.com (L.A.S.); dmitry.filimonov@ibmc.msk.ru (D.A.F.); vladimir.poroikov@ibmc.msk.ru (V.V.P.)

**Keywords:** drug resistance, viral infections, HIV, machine learning, self-consistent regression

## Abstract

Drug resistance of pathogens, including viruses, is one of the reasons for decreased efficacy of therapy. Considering the impact of HIV type 1 (HIV-1) on the development of progressive immune dysfunction and the rapid development of drug resistance, the analysis of HIV-1 resistance is of high significance. Currently, a substantial amount of data has been accumulated on HIV-1 drug resistance that can be used to build both qualitative and quantitative models of HIV-1 drug resistance. Quantitative models of drug resistance can enrich the information about the efficacy of a particular drug in the scheme of antiretroviral therapy. In our study, we investigated the possibility of developing models for quantitative prediction of HIV-1 resistance to eight protease inhibitors based on the analysis of amino acid sequences of HIV-1 protease for 900 virus variants. We developed random forest regression (RFR), support vector regression (SVR), and self-consistent regression (SCR) models using binary vectors containing values from 0 or 1, depending on the presence of a specific peptide fragment in each amino acid sequence as independent variables, while fold ratio, reflecting the level of resistance, was the predicted variable. The SVR and SCR models showed the highest predictive performances. The models built demonstrate reasonable performances for eight out of nine (R^2^ varied from 0.828 to 0.909) protease inhibitors, while R^2^ for predicting tipranavir fold ratio was lower (R^2^ was 0.642). We believe that the developed approach can be applied to evaluate drug resistance of molecular targets of other viruses where appropriate experimental data are available.

## 1. Introduction

The development of viral drug resistance is a serious issue that may decrease the efficacy of drug therapy. The development of drug resistance leading to reduced treatment efficacy has been reported in influenza A virus [1], SARS-CoV-2 coronavirus [2], cytomegalovirus, hepatitis C [3], and human immunodeficiency virus [4,5]. HIV type 1 (HIV-1) leads to progressive immune dysfunction, influences quality of life, and causes the development of infectious and other associated diseases. A large amount of accumulated data on the structural and functional characteristics of the virus and their influence on the development of drug resistance [5] has been collected. Such data can be used to build qualitative and quantitative models of drug resistance. These models can be used either to classify sequences into resistant or susceptible ones or to predict values reflecting the resistance level. The high rate of HIV-1 replication is considered a cause of multiple mutations [5]. Substitutions in the amino acid sequences of HIV-1 proteins necessary for replication and viral particle assembly can directly impact the interaction of a drug with its target, resulting in decreased drug efficacy.

HIV-1 drug resistance can be estimated using phenotypic assays such as PhenoSense and Antivirogram [6]. These methods are based on the exposure of an antiretroviral drug to the HIV-1 variant prevalent in a particular patient with reduced ART efficacy and to a reference wild-type strain at different concentrations. As a result, the fold ratio (FR) value reflecting the level of resistance can be calculated as a ratio between the minimum drug concentration required to inhibit 50% replication of a particular viral variant and the drug concentration required to inhibit 50% of wild-type virus replication [6].

Although phenotypic tests demonstrate high efficacy, they are time-consuming. For these reasons, statistical and machine learning methods were developed to predict resistance based on genotype. Such methods can be used for interpreting and predicting HIV-1 resistance. Various machine learning methods, such as random forest, decision trees, support vector machine, and neural networks, were applied for this purpose [7,8,9,10,11,12,13,14]. Some of these methods were successfully used in HIV-1 drug resistance interpretation systems. For instance, SHIVA uses random forest classification and linear interpolation algorithms to predict phenotype (resistance) based on genotype [13]. Deep learning methods were also applied to predict HIV-1 resistance [10,14].

In the study by N. Beerenwinkel et al., regression models were built to predict resistance factors (fold-change in susceptibility) based on variables indicating a particular amino acid in a certain position of the HIV reverse transcriptase and protease sequences and one 69SS insertion as an additional attribute [12]. The coefficient of determination (R^2^) of the models for protease inhibitors varied from 0.61 (for ATV) to 0.73 (for LPV). The regression models were used as one of the components to build the Geno2Pheno predictive system.

In the study by H. Tunc et al., 2023 [15], the possibility of building models for quantitative prediction of HIV-1 drug resistance based on combined descriptors of the protease and the corresponding inhibitors using artificial neural networks was studied. In total, this study is based on the analysis of over 11,000 values of half-maximal inhibitory concentration changes for eight drugs and more than 1000 HIV-1 variants. The average coefficient of determination (R^2^) of the models obtained as a result of testing when seven of the eight protease inhibitors (PIs) were included in the training set and the remainder in the test set ranged from 0.359 (tipranavir) to 0.822 (lopinavir). The advantage of the developed model is the ability to predict FR for new inhibitors (the mean R^2^ was 0.71). It should be noted that the models in the study by H. Tunc et al. were built using data that included information on the structure of both inhibitors and the target protein sequence (HIV-1 protease). At the same time, the test procedure was based on the exclusion of the structure of inhibitors only. This makes it difficult to estimate the applicability of the models for novel HIV-1 protease sequences as input.

Given the high relevance of predicting drug resistance in viruses, the substantial amount of accumulated data, and the high significance of HIV-1 infection in the disease burden, the aim of our study is to develop models for quantitative prediction of HIV-1 drug resistance using machine learning methods. The application of regression models allows for predicting quantitative resistance values, which are essential for a comprehensive evaluation of drug efficacy in HIV-1 infection treatment and cure for patients with specific virus variants. In our study, the models for quantitative prediction of HIV-1 drug resistance to protease inhibitors are based on the analysis of data on the amino acid sequence of this HIV-1 enzyme.

Based on the developed models, we identified the most significant features involved in the development of drug resistance. Using machine learning methods, HIV-1 protease sequence patterns, significant for resistance development, were determined and compared with the earlier published results.

## 2. Materials and Methods

Genotype–phenotype data were collected from the Stanford University HIV Drug Resistance Database, which contains a large collection of HIV type 1 (HIV-1) isolates (variants) sequences with the values reflecting resistance to the HIV-1 protease, reverse transcriptase, and integrase inhibitors that are typically used in antiretroviral therapy (ART) regimens [16]. The results of drug resistance/susceptibility testing included in the dataset for model building were obtained using the PhenoSense test system [6]. We used genotype–phenotype datasets for eight protease inhibitor (PI) drugs, including fosamprenavir (FPV), indinavir (IDV), nelfinavir (NFV), saquinavir (SQV), lopinavir (LPV), atazanavir (ATV), tipranavir (TPV), and darunavir (DRV). Sequences of variants containing several inaccurately defined amino acid residues at positions of significant drug resistance mutations were excluded because ambiguous information about amino acid composition at these positions may complicate the analysis of the relationship between genotype and phenotype [17]. Sequences containing missing FR values were also excluded. In addition, FR values for duplicated sequences were replaced with median values. As a result, the obtained data set contained information on 900 HIV-1 protease sequences.

The descriptors for the models were represented by binary vectors, which contain 0 or 1 depending on the presence of a specific peptide fragment in each amino acid sequence. The method of splitting the sequence into peptide fragments can significantly affect the quality of the model. The number of unique descriptors is determined by the size of peptide overlap in the sequence. The length of the peptide may affect the quality of the model. To construct descriptors, we use the knowledge of the presence/absence of short peptides consisting of five amino acid residues (this value is chosen empirically) in a given sequence, which allows for generating data sets with the optimal number of descriptors, providing the possibility of obtaining the highest accuracy values for the models. The size of the peptide overlap was also determined empirically: an overlap of two amino acid residues was found to be optimal.

**Figure 1:**
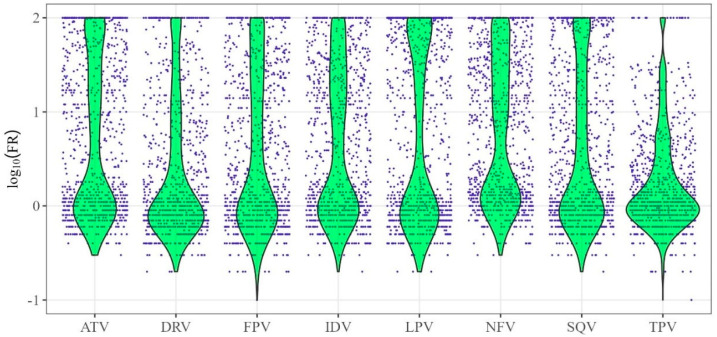
Distribution of logarithmic values of fold rate (log_10_(FR)) for protease inhibitors. The *x*-axis contains the names of HIV-1 protease inhibitor drugs, while the *y*-axis represents the values of log_10_FR—the logarithm of the ratio between the minimum drug concentrations required for 50% inhibition of replication of the analyzed virus and the wild-type virus.

The logarithmic FR values (log10(FR)) values were used to build quantitative models of drug resistance. (Figure 1). Figure 1 shows the distributions of log_10_(FR) values (*y*-axis) for HIV-1 protease inhibitors (*x*-axis).

The distributions of log_10_(FR) values (green color in the figure) are asymmetric and, for some drugs, are significantly shifted toward their minimum and, in some cases, maximum values.

In our study, we applied Random Forest Regression (RFR) [18], Support Vector Regression (SVR) [9,19,20], and Self-Consistent Regression (SCR) [21] algorithms to predict FR for each of the eight drugs using a dataset consisting of 900 observations and 1159 features.

RFR is an ensemble learning method that uses multiple decision trees and combines their predictions to improve accuracy and analyze complex relationships of the data [18]. This method can be effectively used for an analysis of nonlinear relationships between genotype and phenotype and was earlier applied to predicting HIV-1 drug resistance [18].

SVR is a machine learning method based on the Support Vector Machines (SVM) [19]. Numerous studies have validated SVM as an effective tool for predicting HIV-1 drug resistance [9,11,20].

SCR is an in-house developed approach that is aimed at finding significant parameters of models along with relationship evaluation, which can be useful for identifying nonlinear patterns in data [22]. This method is characterized by the high accuracy of models for quantitative prediction of toxicity of drug-like compounds [23,24]. A modified version of this method was used to build classification models of HIV-1 inhibitors for the search for promising HIV-1 inhibitors [25].

The RFR, SVR, and SCR were chosen for the current study because we have previously demonstrated the application of these methods to the analysis of complex structure-property relationships for different types of data [11,20,21,23,24,25].

The best models corresponding to the different approaches were obtained by testing different algorithm parameters. For RFR, hyperparameters such as the number of trees and the maximum number of features considered at each node split were tested; the range of data values for these features was determined empirically. A radial basis function (RBF) kernel was used to build SVR models for all target variables.

For the RFR model, low-significance features were excluded to increase model accuracy. The evaluation of significant variables for model construction utilized a Mean Decrease in Impurity (MDI). MDI measures the total decrease in node impurity (variance) brought by each feature across all trees in the ensemble. The threshold for removing unimportant features was selected empirically based on the model’s performance across different feature subsets. Consequently, a varying number of unique significant descriptors corresponding to pentapeptides were selected for each drug: 454 for FPV; 441 for ATV; 417 for IDV; 346 for LPV; 449 for NFV; 484 for SQV; 623 for TPV; and 521 for DRV. The variances in the set of selected descriptors can be attributed to the different range of importance values assigned to the features when constructing the models of each target variable.

The performance of models was estimated using the coefficient of determination (R^2^) and root mean square error (RMSE) calculated by cross-validation:

(1) $$R^{2}=\frac{\sum_{i=1}^{n}{\left({\hat{y}}_{i}-\bar{y}\right)}^{2}}{\sum_{i=1}^{n}{\left(y_{i}-\bar{y}\right)}^{2}}$$

(2) $$RMSE=\sqrt{\frac{\sum_{i=1}^{n}{\left({\hat{y}}_{i}-y_{i}\right)}^{2}}{n}}$$

The predictive performance of the models was evaluated using a five-fold cross-validation, which is performed on the entire dataset and is based on dividing the entire dataset into 5 subsets, with one subset representing the test set.

## 3. Results

The characteristics of the quantitative models for prediction of the HIV-1 drug resistance with the best predictive ability are presented in Table 1. The performance of the RFR model after the removal of the features with low significance is provided. The most substantial increase in accuracy after removing insignificant features was observed for the drugs saquinavir, tipranavir, and darunavir.

The values of R^2^ and RMSE are, in general, comparable for all methods in our study. For most drugs, the accuracy of the models built using SCR and SVR exceeded that of the RFR model. The R^2^ values for the DRV and TPV were higher for the SVR compared to the SCR method. The performance of the models varies depending on the specific drug to which resistance is predicted. This may be due to specific substitution patterns associated with higher levels of resistance to a particular drug. In addition, the predictive ability of the models is obviously influenced by the original experimental data (Figure 1) and probably also by the amount of accumulated data on the genotype–phenotype relationship for each drug.

**Figure 2:**
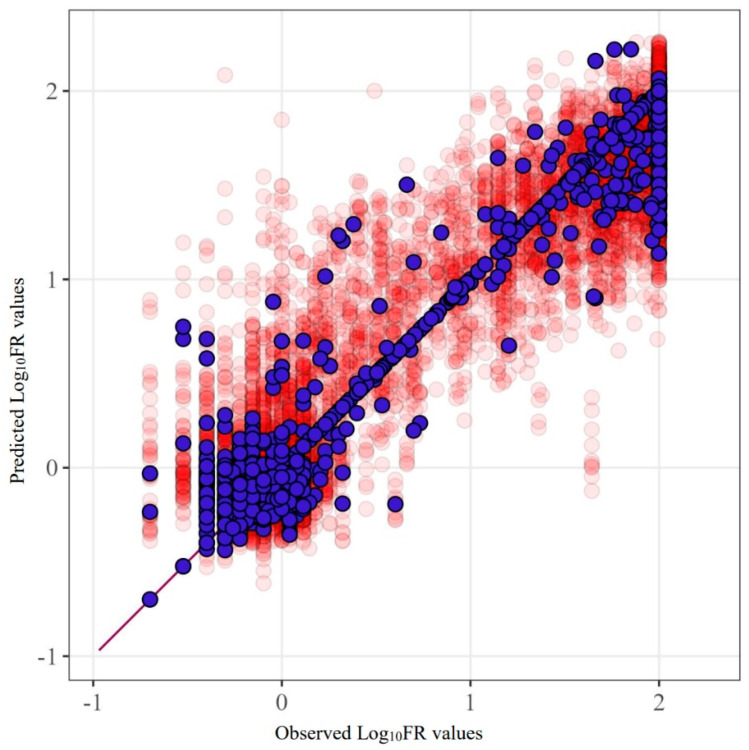
Predicted and observed values for the FPV model built using SCR. Purple markers represent the model’s predictions before excluding test sets from the data, while red markers show the results of five-fold cross-validation.

Figure 2 provides an example of the SCR prediction for the drug fosamprenavir (R^2^ = 0.842). This figure clearly demonstrates the characteristics of the data used and their influence. The main errors are concentrated in proximity to the extreme values of the quantities.

The lowest accuracy of the model was obtained for tipranavir for all methods. In contrast, fosamprenavir, lopinavir, and darunavir show relatively high prediction accuracy.

## 4. Discussion

The relationships between the metrics of the model’s predictive ability for different drugs and the variations in the mutation patterns described for each drug were analyzed. We compared our results with data from a study that searched for significant mutations for eight protease inhibitor drugs. In this work, 11 mutations were found for fosamprenavir (L10F, V32I, L33F, M46IL, I47AV, I50V, I54LM, L76V, V82F, I84AV, I154ST), 15 for atazanavir (M46L, G48V, I50L, I54V, G73ST, I84AV, N88S, L90M, L24I, G48M, F53L, I54AMST, G73C, V82S, N88D), 17 for indinavir (L10F, L24I, V32I, M46IL, I54V, G73S, L76V, V82ATF, I84AV, N88S, L90M, I47A, G48M, I54ATS, G73CT V82S), 15 for nelfinavir (L10FI, D30N, M46IL, G48V, I54V, G73S, V82F, I84AV, N88DS, L90M, L24I, V32I, I54AST, G73T, V82S), 9 for saquinavir (G48V, F53L, I54TV, G73S, I84AV, L90M, G48M, I54AS, G73T), 13 for lopinavir (L10F, L24I, V32I, L33F, M46IL, I47AV, I50V, I54LMV, L76V, V82AFST, I84AV, G48M, I54AST), 8 for tipranavir (L33F, K43T, I47V, I54AMV, T74P, V82LT, N83D, I84V), and 9 for darunavir (V32I, L33F, I47V, I50V, I54LM, T74P, L76V, I84VA, L89V) [25]. The largest number of described mutations was characteristic of ATV and IDV drugs, which explained the high accuracy of model predictions in predicting FR for these drugs. For SQV, TPV, and DRV drugs, half as many mutations were described that increased sensitivity to these inhibitors. The quality of the experimental data, therefore, limits the predictive power of the models built for these drugs.

By evaluating the Mean Decrease in Impurity, an embedded parameter that describes feature importance in Random Forest Regression, we identified the top twenty features (the number of features was selected empirically) with the highest significance for each drug when building the regression model in our study and compared them with known substitutions in HIV-1 protease. Significant features in our model correspond to unique pentapeptides in the HIV-1 protease sequence that influence the prediction of the FR value. We found that the selected significant peptide fragments corresponded to intervals of positions in the protease sequence, specifically intervals of positions 7–11, 10–14, 31–35, 43–47, 46–50, 52–56, 67–71, 70–74, 79–83, 82–86, and 88–92, which were determined to be significant in the prediction of FR for the majority of protease inhibitors investigated. Notably, peptide fragments that were selected using our approach contain various amino acid substitutions at the same position of the HIV-1 protease amino acid sequence. For example, peptides TIFEE and TVLEE (31–35) contain mutation V32I, KPKMI (42–46); MIGGI and IVGGI (46–50) contain I47V; GFIKV and GFVKV (52–56)—I54V; PTPVN and PTPAN (79–83)—V82A; VNIIG and VNVIG (82–86)—I84A; NVMTQ and NLLTQ (88–92)—L90M. According to the study by Rhee S. Y. et al., mutations associated with increased sensitivity to protease inhibitors include D30N, V32I, I47A, G48VM, I50VL, I54ALMSTV, L76V, V82FSTAL, I84AV, N88S, and L90M [25]. Thus, 7 of the 11 intervals found in our research contain mutation positions that were confirmed by the results of other researchers. In addition, we found four new mutation patterns (positions 7–11, 10–14, 67–71, 70–74) that were not previously described in the literature and may contribute to resistance development.

We compared our results with those from the Geno2pheno predictive system (Table 2), which is aimed at building regression models for predicting values of Log10 fold resistance (the so-called resistance factors) of HIV protease and reverse transcriptase sequences [12]. This study is one of the few quantitative predictive models known from the literature, characterized by a reasonable accuracy of prediction and based on sequence data features. In the work by Beerenwinkel N. et al. [12], the R^2^ for the SVR model for predicting resistance factors to HIV-1 protease inhibitors varied from 0.61 (MSE = 0.262) to 0.73 (MSE = 0.204) obtained in 10-fold cross-validation. The mean squared errors (MSE) ranged from 0.169 to 0.262. Specifically, the highest R^2^ values were obtained for IDV and LPV. Overall, taking into account differences in the validation, the R^2^ values of our models are, in general, comparable to those from the previously published study [12], while for some drugs (ATV, NFV), the R^2^ values of our models are slightly higher.

It should be noted that an exact comparison of the results of the present study and the study by H. Tunc et al. [15] is difficult. These difficulties arise due to differences in the study design (i.e., models aimed at predicting HIV drug resistance to known inhibitors based on protein sequence (our models) and predicting HIV drug resistance to novel inhibitors based on both protein and inhibitor structure [15]). Validation procedures (5-fold cross-validation in our study, exclusion of inhibitor structures, and creation of a custom test set [15]) are also different. In contrast to the models described in the study by H. Tunc et al. in 2023 [15], our models were built without the use of drug structure descriptors. The determination coefficients (R^2^) of our models (SCR, SVR) are comparable with those obtained by H. Tunc et al. For darunavir, saquinavir, and tipranavir, the R^2^ in the study by H. Tunc is slightly higher than in our study when the entire data set is used for validation. However, the performances of SCR and SVR models for lopinavir and tipranavir are, in general, insignificantly higher. Therefore, it seems that for quantitative prediction of drug resistance, knowledge of the primary structure of HIV-1 enzymes is sufficient to develop models with reasonable performance.

A more accurate comparison of model quality requires a study using identical test samples, which is challenging due to differences in study design and objectives. It is important to note that RMSE (MSE) values may not be suitable for evaluating performance in the context of FR prediction because the FR values may vary significantly [25]. Consequently, even logarithmic values can vary significantly within a single data set, as well as between different drugs.

The applicability of the models presented in our study is limited to cover sequences of HIV variants (isolates) that do not contain multiple unresolved amino acid residues (the so-called “mixtures”), especially in the major drug resistance positions for which the association with the development of drug resistance has been widely studied.

## 5. Conclusions

In the present study, we investigated the possibility of building models for quantitative prediction of drug resistance of viruses, with HIV as a case study. We built models based on the amino acid sequences of the structural enzyme protease. The performance is comparable for models based on SVR and SCR, with slightly lower values of R^2^ and RMSE obtained for RFR. The predictive performance (R^2^, RMSE) may vary depending on the drug, the level of resistance to which has been predicted.

The results of our study are comparable to previously reported experiments. The performance of the models is limited by the peculiarities of the experimental data, which are characterized by significant differences. The best results of regression models can be obtained for drugs for which high-quality data on the “genotype–phenotype” or, in other words, “structure–resistance” relationship have been accumulated.

Nevertheless, the possibility of modeling the level of decrease in the half-maximal inhibitory concentration of a drug for individual variants of viruses suggests that the analysis of the “structure–resistance” relationship is possible not only for qualitative models but also for models of quantitative analysis of drug resistance of viruses with the availability of experimental data [26].

## Figures and Tables

**Table 1:** Results of quantitative prediction (coefficient of determination, R^2^, and root mean square error, RMSE) of the outcome variable for the logarithm of the reduction in half-maximal inhibitory concentration for the corresponding drug (log_10_FR).

Drug Name	RFR, R^2^	RFR, RMSE	SVR, R^2^	SVR, RMSE	SCR, R^2^	SCR, RMSE
FPV	0.843	0.731	0.858	0.693	0.842	0.701
ATV	0.855	0.717	0.872	0.674	0.873	0.661
IDV	0.851	0.675	0.857	0.669	0.850	0.682
LPV	0.912	0.619	0.909	0.632	0.908	0.630
NFV	0.836	0.692	0.852	0.662	0.850	0.663
SQV	0.793	0.844	0.829	0.756	0.832	0.749
TPV	0.593	0.754	0.642	0.704	0.559	0.798
DRV	0.806	0.726	0.828	0.678	0.802	0.705

**Table 2:** Comparison of the predictive metrics of the models presented in this study with previously published approaches.

Drug Name	SCR, R^2^, 5-Fold CV *	SCR, RMSE, 5-Fold CV *	R^2^, SVR, 5-Fold CV *	SVR, RMSE, 5-Fold CV *	R^2^, SVR, 10-Fold CV ** [12]	MSE, SVR, 10-Fold CV ** [12]
FPV ***	0.842	0.701	0.859	0.693	-	-
ATV	0.873	0.661	0.872	0.674	0.61	0.262
IDV	0.850	0.682	0.857	0.669	0.73	0.197
LPV	0.908	0.630	0.909	0.632	0.73	0.169
NFV	0.850	0.663	0.852	0.662	0.71	0.207
SQV	0.832	0.749	0.829	0.756	0.71	0.204
TPV	0.559	0.798	0.642	0.704	-	-
DRV	0.802	0.705	0.828	0.678	-	-
APV ***			-	-	0.65	0.173

* R^2^, RMSE obtained in our study using five-fold cross-validation. ** R^2^, MSE according to the values published by N. Beerenwinkel et al. [12] using ten-fold cross-validation used in the study by N. Beerenwinkel et al. [12]. *** FPV is a phosphate prodrug of APV.

## Data Availability

The datasets and underlying code generated and analyzed during the current study are available in the following repository: https://github.com/ekaterinastolbova/Quantitative-Prediction-of-HIV-Drug-Resistance, accessed on 10 July 2024.
